# Neural representation of spectral and temporal features of song in the auditory forebrain of zebra finches as revealed by functional MRI

**DOI:** 10.1111/j.1460-9568.2007.05865.x

**Published:** 2007-11

**Authors:** Tiny Boumans, Frédéric E Theunissen, Colline Poirier, Annemie Van Der Linden

**Affiliations:** 1 Bio-Imaging Laboratory, University of Antwerp Belgium; 2 Helen Wills Neuroscience Institute and Psychology Department, University of California Berkeley, CA, USA

**Keywords:** anaesthesia, auditory cortex, birdsong, *Taeniopygia guttata*, vocalization

## Abstract

Song perception in songbirds, just as music and speech perception in humans, requires processing the spectral and temporal structure found in the succession of song-syllables. Using functional magnetic resonance imaging and synthetic songs that preserved exclusively either the temporal or the spectral structure of natural song, we investigated how vocalizations are processed in the avian forebrain. We found bilateral and equal activation of the primary auditory region, field L. The more ventral regions of field L showed depressed responses to the synthetic songs that lacked spectral structure. These ventral regions included subarea L3, medial-ventral subarea L and potentially the secondary auditory region caudal medial nidopallium. In addition, field L as a whole showed unexpected increased responses to the temporally filtered songs and this increase was the largest in the dorsal regions. These dorsal regions included L1 and the dorsal subareas L and L2b. Therefore, the ventral region of field L appears to be more sensitive to the preservation of both spectral and temporal information in the context of song processing. We did not find any differences in responses to playback of the bird's own song vs other familiar conspecific songs. We also investigated the effect of three commonly used anaesthetics on the blood oxygen level-dependent response: medetomidine, urethane and isoflurane. The extent of the area activated and the stimulus selectivity depended on the type of anaesthetic. We discuss these results in the context of what is known about the locus of action of the anaesthetics, and reports of neural activity measured in electrophysiological experiments.

## Introduction

Auditory perceptual categories are coarsely divided into loudness, rhythm or tempo, pitch (voicing and prosody in speech) and timbre (formants in speech), and it has been postulated that separate neural systems implementing different computations might underlie these different percepts. Indeed, neuroimaging and neurological studies of human music performance, perception and comprehension have shown that neural systems underlying music and speech processing are distributed throughout the left and right cerebral and cerebellar hemispheres, with different aspects of music processed by distinct neural circuits (Liegeois-Chauvel *et al.*, 1998; Zatorre, 2001; Griffiths, 2003; Kohlmetz *et al.*, 2003; Di Pietro *et al.*, 2004; Murayama *et al.*, 2004; Schmithorst, 2005).

A large number of studies has also shown specialization at the level of single neurons for acoustical features underlying different perceptual attributes (reviewed in Eggermont, 2001). In the neurophysiological literature, the computations are often described in terms of extracting spectral (or textural) features of sounds vs temporal (or contour) features of sounds, with the underlying assumption that spectral features are essential for pitch and timbre percepts, and temporal features are essential for rhythm but also timbre as well. The division between spectral and temporal features is fundamental for many reasons. First, from an acoustical engineering standpoint, extracting temporal features requires broad frequency filters with fast time constants, whereas extracting spectral features requires narrow frequency filters with long time constants (Cohen, 1995). Second, the vertebrate peripheral auditory circuitry appears to rapidly divide into branches specialized either for temporal processing or specialized for intensity and spectral processing (Young, 1998; Cant & Benson, 2003). Similar specializations might be maintained or recreated along with others at the levels of the primary and secondary auditory cortex (Bendor & Wang, 2005). Third, a statistical analysis of human and animal vocalizations shows that spectral and temporal acoustical features are distinctly separate: animal vocalizations are primarily composed of short sounds with little spectral structure (e.g. consonants), and longer sounds with a rich spectral content (e.g. voiced vowels; Lewicki, 2002; Singh & Theunissen, 2003; Cohen *et al.*, 2006).

For these reasons, we postulated the presence of distinct auditory forebrain areas specialized for processing temporal information vs spectral information. We were also motivated by the desire to bridge the gap between auditory research using imaging techniques in humans, which has been best suited to identifying putative neural regions that mediate different perceptual acoustical attributes; and auditory research in animal models, which has best described the underlying neural computations (Griffiths *et al.*, 2004). We tested our hypothesis in a well-established animal model for understanding the neural basis of vocal learning and the perception of vocalizations, the songbird (reviewed in Theunissen & Shaevitz, 2006). The auditory forebrain regions in songbirds have been shown to be specialized for processing natural sounds and song in particular (Grace *et al.*, 2003; Hsu *et al.*, 2004; Woolley *et al.*, 2005). In addition, neurons found in brain regions involved in song production and learning (the song nuclei) show a remarkable degree of selectivity for the bird's own song (BOS; Margoliash, 1983, 1986; Doupe & Konishi, 1991). The neural recognition of the BOS in the song nuclei requires the preservation of both the spectral and temporal structure of song (Theunissen & Doupe, 1998). In this study, we used blood oxygen level-dependent (BOLD) functional magnetic resonance imaging (fMRI) in the zebra finch to examine whether different regions of the songbird auditory forebrain are specialized for extracting temporal vs spectral features found in songs.

## Materials and methods

### Subjects

Seven adult male zebra finches (*Taeniopygia guttata castanotis*) served as subjects for this experiment. Experimental procedures were in agreement with the Belgian laws on the protection and welfare of animals, and had been approved by the ethical committee of the University of Antwerp (Belgium).

The anaesthetized birds were immobilized in a non-magnetic, custom-made head holder composed of a beak mask and a circular radio-frequency (RF) surface antenna (15 mm) pushed down around the bird's head above both ears and eyes. Body temperature was continuously monitored with a cloacal temperature probe (SA-Instruments, New York, USA) and maintained at 40 °C by a feedback-controlled heating system (SA-Instruments). Respiration rate and amplitude were constantly monitored with a small pneumatic sensor (SA-Instruments) positioned under the bird.

#### Anaesthetic

Medetomidine, isoflurane and urethane were used alternatively as anaesthetic. The effect of anaesthesia both on the neural response and on the transfer function between the neural activity and the BOLD response measured in fMRI is a continuing and important research topic. It has been shown that anaesthetic level can profoundly change the BOLD response both because of changes in neural activity (Richards, 2002) and because of changes in the BOLD transfer function (Masamoto *et al.*, 2007). Medetomidine, a potent α2-adrenoreceptor agonist, was chosen because it had been used previously with good results in the first functional imaging study in songbirds (Van Meir *et al.*, 2005). Medetomidine has also been shown to be an effective non-toxic alternative to α-chloralose anaesthesia in fMRI studies in the somatosensory and visual cortex of rats (Van Camp *et al.*, 2006; Weber *et al.*, 2006). Urethane is a popular anaesthetic in neurophysiological studies in small animals because of its relatively small effect on the neural response (Maggi & Meli, 1986). Urethane has been used extensively in neurophysiological investigations of the avian auditory and vocal systems (reviewed in Theunissen *et al.*, 2004), and its use allows us to compare, more directly, our results with that in the literature. Finally, isoflurane is the most common anaesthetic in clinical applications and it has also been used in most of the imaging studies with monkeys and cats (Logothetis *et al.*, 1999; Kim *et al.*, 2000; Duong *et al.*, 2001). Isoflurane has the great advantage of having relatively minor side-effects and, for this reason, could have the most potential for longitudinal studies in songbirds.

All birds underwent fMRI measurements under medetomidine (*n* = 7), and some of the same birds were measured with isoflurane (*n* = 3) and/or urethane (*n* = 4) anaesthesia. For medetomidine, birds initially received an intramuscular injection in the pectoral muscles of 25 mg/kg ketamine (Ketalar, 50 mg/mL, Parke-Davis, Belgium) and 2 mg/kg medetomidine (Domitor, 1 mg/mL, Orion Pharma, Finland). After 30 min, medetomidine was continuously infused at a rate of 0.03 mL/h through a catheter positioned in the chest muscle. Isoflurane was administered through a small mask over the bird's beak at a flow rate of 200 cc/min oxygen and 400 cc/min nitrogen. The concentration of isoflurane was 3% during the induction of anaesthesia and 1.5% during functional imaging. Urethane anaesthesia was administered with three 20-µL shots (20% concentration) separated by 30 min each.

### Auditory stimuli and stimulation

#### BOS recording

The song of each individual bird was recorded in the laboratory of J.J.Bolhuis. The birds were placed individually in soundproof isolation chambers (115 × 115 × 205 cm) to record their undirected songs. Recordings were made using a Sennheiser MKH50 P48 microphone (Sennheiser Electronic KG, Wedemark, Germany) and a PC with Avisoft Recorder software (Berlin, Germany). All bird sounds were automatically recorded for approximately 20 h. For each bird a stereotypical song of approximately 2 s was selected. This song was then repeated to build a long song bout of 2 min. The silence interval between the songs within the 2-min song bout was chosen from a uniform distribution between 330 ms and 1 s (average 660 ms). This intersong interval was typical of the intervals between songs in the bouts of undirected song that we recorded. The power of the songs from each bird was normalized to have equal root mean square power. Conspecific stimuli used in the experiments also came from these recordings. For each bird, the stimulus ensemble included the BOS, multiple familiar conspecific songs (CON), and spectrally filtered (CON-sf) or temporally filtered (CON-tf) versions of these CONs.

#### Synthesis of filtered song stimuli

**Fig. 1:**
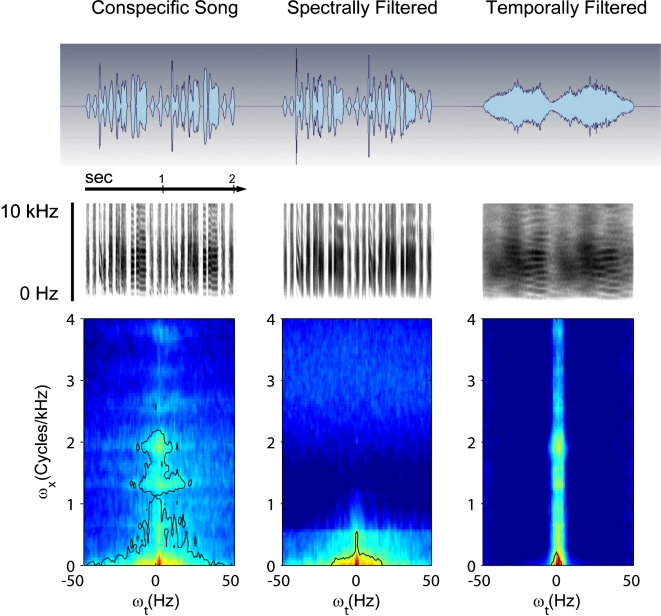
Oscillograms (top row), spectrograms (middle row) and modulation spectra (bottom row) showing the spectral and temporal features found in the experimental auditory stimuli. The figure shows an example of conspecific song before (CON) and after spectral (CON-sf) and temporal filtering (CON-tf). The modulation spectrum (see online edition for colour figure) quantifies the spectrotemporal structure that is present in the sound (see Material and methods). ω_x_ = spectral modulations, ω_t_ = temporal modulations.

The synthetic songs were obtained by low-pass filtering the natural songs' temporal or spectral modulations. This filtering operation was performed in the space of the modulation spectrum and should not be confused with more typical frequency filtering operations. The modulation spectrum is obtained by performing a 2D power spectrum of a time–frequency representation of the sound, in our case the log of the spectrogram (Singh & Theunissen, 2003). The modulation spectrum of a particular song is shown in Fig. 1 (bottom row, left panel). The *x*-axis represents the temporal amplitude modulation frequencies in the narrowband signals obtained by a decomposition of the sound in different frequency bands as performed in a spectrogram. The *y*-axis represents the spectral modulations of the same amplitude envelopes but across frequency bands, in units of cycles/kHz. The colour of the modulation spectrum (see online edition) codes the energy of modulations − as a function of joint temporal modulations and spectral modulations. The logarithm of the modulation spectrum is used to disentangle multiplicative spectral or temporal modulations into separate terms. For example, in speech sounds, the spectral modulations that constitute the formants in vowels (timbre) separate from those that constitute the pitch of the voice. For natural sounds, the modulation spectrum follows a power law relationship with most energy concentrated at the low frequencies. For zebra finch (as well as other animal) vocalizations, most of the temporal modulations in the envelope are found below 25 Hz. Most of the energy in the spectral modulations of animal vocalizations is found below 2.5 cycles/kHz (Singh & Theunissen, 2003; Cohen *et al.*, 2006). Finally, as mentioned in the Introduction, the modulation spectrum of animal vocalizations including human speech shows a degree of inseparability: vocalizations are made of short sounds with little spectral structure but fast temporal changes (found along the *x*-axis at intermediate to high temporal frequencies), and slow sounds with rich spectral structure (found along the *y*-axis at intermediate to high spectral frequencies).

The filtering operation that we performed here was designed to generate songs with equal power and equal overall frequency power spectra as the original song but lacking either the natural temporal modulations or the natural spectral modulations. For this purpose, we low-pass filtered the song in the space of the modulation spectrum. For each song we first generated a spectrogram and took its logarithm. We then calculated the joint spectral and temporal amplitude spectrum (also called the modulation spectrum) and the phase spectrum by taking the 2D fast Fourier transform of the log spectrogram. We preserved the modulation phase but low-pass filtered the modulation spectrum to remove either temporal structure or spectral structure. The temporal modulation frequency cut-off was set at 3 Hz (i.e. amplitude envelope changes faster than 3 Hz were filtered out). The spectral modulation frequency cut-off was set at 0.5 cycles/kHz (i.e. spectral structure within any 2-kHz band is filtered out, and thus pitch below 2 kHz is filtered out). A smooth cut-off value was implemented with a cosine square ramp in the filter gain of 1 Hz for temporal modulation filtering and 0.1 cycles/kHz for spectral modulation filtering. After low-pass filtering the modulation amplitude and preserving the phase, an inverse 2D fast Fourier transform and exponentiation was used to obtain a filtered spectrogram. In order to obtain a sound pressure wave function, this spectrogram was then inverted using a recursive algorithm (Griffin & Lim, 1984). The initial choice of time–frequency scale in the spectrographic representation affects both the upper limits of resolvable temporal and spectral modulations, and the efficiency of the spectrum inversion algorithm (Singh & Theunissen, 2003). We used spectrograms made with 128-Hz frequency-width Gaussian filters for the spectral filtering and 32-Hz frequency-width for the temporal filtering. Either of those time–frequency scales captured most of the spectro-temporal structure in song (i.e. the resulting waveforms yielded very similar modulation spectra) but also preserved the efficiency of the spectrum inversion. The entire filtering operation was implemented in Matlab (MathWorks, MA, USA) with routines created by Frédéric Theunissen. The software is available upon request.

Figure 1 shows the oscillograms, spectrograms and modulation spectra of the CON-sf and CON-tf song. The spectrally filtered song has effectively an identical oscillogram as the overall temporal modulations in the amplitude envelope have been preserved. On the other hand, the spectral envelope has been smeared as illustrated in the spectrogram. The CON-sf song is similar to a synthetic sound obtained by amplitude-modulating coloured noise using the temporal envelope of the song, with the exception that CON-sf additionally preserves local spectral quality. It therefore resembles the synthetic temporal speech sounds used by Shannon (Shannon *et al.*, 1995). The temporally filtered song preserves the notes and other harmonic sounds found in zebra finch song (as shown in the spectrogram) but smears the envelope, leaving only the slowest amplitude modulation changes (as shown in the oscillogram).

#### Stimulation protocol

All birds were exposed to four different acoustic signals in pseudo-random order. The pseudo-random order was generated by randomly choosing a starting CON stimulus type, followed by a stimulus determined by transition probabilities. Following CON, we played either the corresponding CON-tf (*P* = 1/3) or the corresponding CON-sf (*P* = 1/3) or the BOS (*P* = 1/3). Following CON-tf or CON-sf, the previous CON was always repeated. Following BOS, a new CON was chosen pseudo-randomly (i.e. without replacement until the list was exhausted). To have sufficient variability of CONs and to ensure that both BOS and different CON were presented an approximately equal number of times, each stimulation protocol contained the familiar songs of five different birds. Our stimulation protocol also allowed pair-wise comparisons of the differential responses to CON and its spectrally or temporally filtered version as CON and a filtered version of CON were always presented in temporal succession and in equal numbers.

Auditory signals were presented to the birds with magnetless dynamic speakers, as described in Van Meir *et al.* (2005). Stimulus application was controlled by Presentation software (Neurobehavioral Systems, version 0.76, Albany, CA, USA). Images were collected with a block-design paradigm consisting of 30 cycles of eight images collected during stimulation and eight images collected during rest, resulting in 480 functional images. Imaging was triggered to the stable respiration periods to minimize fluctuating artefacts. As a consequence, the duration of each stimulation and rest period was not fixed, but ranged from 30 s to 1 min. Each experiment, which was preceded by the acquisition of nine dummy images to allow the signal to reach a steady state, thus took at least 30 min. Up to three consecutive experiments were performed during which the birds were exposed multiple times to the four different stimuli (BOS, CON, CON-sf, CON-tf) in pseudo-random order as described above.

#### Sound levels

The average song power was set at 65 dB sound pressure level (SPL). The song power of the loudest syllable (maximum song power within a 50-ms window) was about 70 dB SPL. The magnet noise (see below) was measured to be about 63 dB SPL (see below). All sound levels were measured inside the magnet with a magnetic electret microphone.

### fMRI experiments

#### Imaging settings

Magnetic resonance (MR) imaging was performed at 300 MHz on a 7 Tesla horizontal bore NMR microscope with an actively shielded gradient-insert (Bruker BioSpin MRI GmbH, Ettlingen, Germany), having an inner diameter of 90 mm and a maximum gradient strength of 300 mT/m. A Helmholtz (45 mm) and a circular RF surface antenna (15 mm) served for transmitting and receiving the RF pulses, respectively.

A set of one sagittal, one horizontal and one coronal gradient-echo (GE) scout image, and a set of 12 horizontal fast GE images were first acquired to determine the position of the brain in the magnet.

Functional imaging was performed using a T2*-weighted single-slice GE fast low-angle shot (FLASH) sequence [field of view (FOV) = 25 mm, echo time (TE) = 14 ms, repetition time (TR) = 40 ms, flip angle = 11°, gradient ramp time = 1000 µs, acquisition matrix = 128 × 64, reconstruction matrix 128 × 128, slice thickness = 0.5 mm]. Long gradient ramp times (1000 µs instead of 100 µs) were used to reduce the gradient noise.

**Fig. 2:**
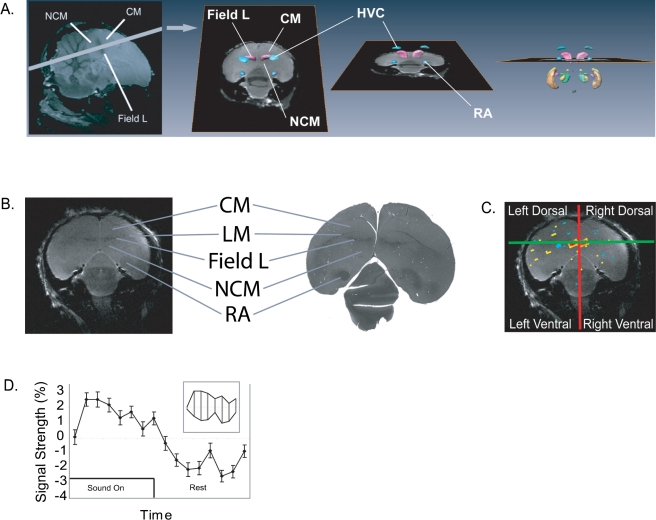
(A) Imaging slice position. The fMRI slice is a tilted coronal-horizontal slice that went through primary auditory pallium (field L), secondary auditory pallium [caudal medial nidopallium (NCM), CLM, CMM] and song control robust nucleus of the arcopallium (RA). The imaging slice is displayed in a 3D rendering volume of zebra finch brain with a coloured representation (see online edition for colour figure) of some key auditory regions and song control nuclei (Poirier *et al.*, in preparation). The region in pink corresponds to field L; the regions in blue to song nuclei HVC and RA; the region in green to area X; and the region in yellow to the ectostriatum. These areas were delineated in a T2-weighted 3D spin-echo dataset of the head of a perfused zebra finch that was postfixed in a mixture of paraformaldehyde (4%) and Dotarem (1%), a paramagnetic MR contrast agent. (B) Comparison between *in vivo* anatomical MR image and *in vitro* histological slice in silver stain with same orientation. The darker horizontal band seen in the structural MR image corresponds to the dense fibre track that defines subregion L2a. The song control nucleus RA can be seen in both the structural MR image and in the silver stain. The border between the nidopallium and the mesopallium as determined by the lamina mesopallialis (LM) is seen in the silver stain and can be detected in the structural MR image. This landmark can be used to determine whether the observed activity was in field L or the secondary auditory area, CM. On the other hand, the actual border between NCM and the primary auditory pallium cannot be discriminated. (C) Regional analysis. The auditory regions are divided into four quadrants determined by the midline dividing the right from left hemisphere, and determined by the horizontal fibre track dividing ventral from dorsal regions. The dorsal quadrants include the dorsal part of L2a, L1 and L/L2b, while the ventral quadrants include the ventral part of L2a, L3, the medial-ventral part of L and NCM.(D) Time series BOLD signal. The curve shows the average time course of the BOLD signal for a single pixel that had a highly significant response. The first eight images are acquired during stimulation followed by eight images acquired during rest. Each time tick is approximately 5 s (see Materials and methods). The strength of the signal was the point-by-point difference between the signal during stimulation and the signal during rest as illustrated in the inset figure. It is also the area between these curves. The last points in the curve were excluded if their contribution added more noise than signal (see Materials and methods).

As illustrated in Fig. 2, the functional images were acquired on a tilted coronal slice that was chosen to intersect all critical auditory forebrain areas: the primary auditory region field L, as well as secondary auditory areas caudal medial nidopallium (NCM) and caudal mesopallium (CM), both of which have been implicated in the processing of familiar songs (reviewed in Bolhuis & Gahr, 2006). The angle of the slice was determined by first finding the tangent line that touched the back of the cerebellum and the top of the cerebrum. That tilted line was further rotated towards the horizontal by 10 °. The line was then moved rostrally by 2.5 mm.

Anatomical high-resolution imaging was performed at the same position as the functional imaging slice with a T2-weighted fast spin-echo (RARE) sequence (FOV = 25 mm, TE = 14 ms, effective TE = 56.88 ms, TR = 2800 ms, acquisition matrix = 256 × 256, slice thickness = 0.5 mm, RARE factor = 8, and four averages).

#### Image processing

All image processing was performed in Matlab with custom-written software. The raw time series for each pixel were first thresholded at a level determined by a histogram of signal strengths in order to separate signal in brain regions from the signal in non-brain regions. All operations were then performed on this thresholded signal without further spatial or temporal smoothing. The spatial resolution of the pixels was about 200 µm square (FOV = 25 × 25 mm, number of pixels 128 × 128). The time between images was variable, as image acquisition was performed only during stable phases of the respiratory pattern, and ranged from 3.75 s to 7.5 s. Most of the variability in the interimage acquisition time was across birds or experiments.

The time series consisted of the succession of the eight time points acquired during stimulation followed by the eight time points acquired during rest (see above and Fig. 2). A difference time signal was then calculated by subtracting the two time curves from each other point by point. Then eight average signal differences were estimated by summing these difference curves for one time point, two time points, and so forth until the entire difference signal was summed. Eight statistical tests of significance at each pixel (one sample *t*-test) were then performed for each of these eight average signal differences. The number of time points in the sum that gave the highest significance over all pixels was then used to calculate the signal strength. The rationale for this procedure is that the observed BOLD signal was characterized by both an increase during stimulation and a decrease during rest (see Fig. 2D). Moreover, both the increase and decrease started (and often peaked) at the very first time point but then, after the first or second time point, decreased monotonically to baseline, often before the end of the eight images. Our simple procedure was designed in order to maximally detect this characteristic signal without adding the noise found at the end of the time trace. An average signal for each significant pixel was obtained by averaging over all time points. The reported signal strength is then the best average signal difference divided by the average signal. All non-significant pixels and all isolated statistically significant pixels were deleted from the analysis. Furthermore, as described in more detail in the Results, we performed most of our analysis on a region of interest (ROI) defined by the large contiguous region of activity centred around the primary auditory region.

## Results

The principal goal of this study was to investigate whether temporal and spectral information of complex sounds were processed in separate auditory regions of the songbird brain. To answer this question, we used recently developed BOLD imaging techniques for small animals − songbirds in particular. The BOLD response gives a coarse representation of the neural activity, but has the advantage that activity in the entire brain (or large parts of the brain) can be measured simultaneously. Because our study is to date one of the first studies of neural activity in birds using the BOLD fMRI technique, we also investigated the effect of different anaesthetics on the response. Our study is also the first quantitative assessment of the distribution of the auditory evoked BOLD response in the zebra finch forebrain.

### Localization of BOLD responses

We chose to visualize a tilted coronal-horizontal slice in order to sample most of the auditory regions in the zebra finch forebrain, as previously determined in anatomical and physiological experiments. Our slice went through primary auditory region field L (including subareas L1, L2, L3 and L) and secondary auditory (or associative) regions caudal medial mesopallium (CMM), caudal lateral mesopallium (CLM) and NCM. The slice also went through the song robust nucleus of the arcopallium (RA).

**Fig. 3:**
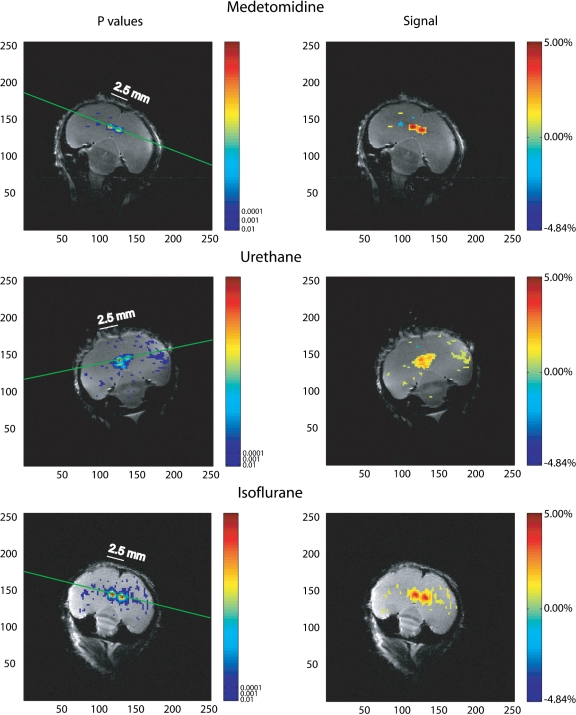
Average BOLD signal and anaesthetic (see online edition for colour figure). The images illustrate the typical activation patterns that were found under the three anaesthetics used in the experiment: medetomidine (top row), urethane (middle row) and isoflurane (bottom row). The signal shown here is for all sounds presented and all brain images averaged together. The left column shows the *P*-values of significant activated pixels, the right column shows the signal strength relative to the mean signal difference. The green line shows the dorso-ventral axis. Bilateral activation of the primary auditory pallium (field L) from the midline up to approximately 1.5 mm lateral (range: 0.8–2.3 mm) can be observed. Urethane and isoflurane show larger activation areas than medetomidine. With urethane and isoflurane, a second pole of activity is sometimes seen on the medial and ventral side of the nidopallium.

For all sound stimuli and in all anaesthetic conditions, we found strong and reliable symmetric bilateral activation of the primary auditory region. Figure 3 shows typical examples of activation for the three anaesthetics used in the experiment, for all sounds, averaged together. Bilateral activation of field L from the midline up to approximately 1.5 mm lateral could be seen in all the acquired images (range: 0.8–2.3 mm). The average medial to lateral extent was 1.48 mm with no statistical differences between the right and the left side of the brain (right side: 1.462 mm; left side: 1.493 mm; two-tailed paired *t*-test *P* = 0.81). The peak of the BOLD signal was found on average at 689 ± 42 microns from the midline with again no statistical differences between the right and left side (right side: 670 µm; left side: 700 µm; two-tailed paired *t*-test *P* = 0.81). The extent of activity in the tilted dorsal-ventral axis was about 1.57 mm and was also symmetric (right side: 1.568 mm; left side: 1.58 mm; two-tailed paired *t*-test *P* = 0.88). The peak of the bilateral activity was almost always in precise register with the darker horizontal band in the structural MR image that corresponds to the dense fibre track defining subregion L2a as shown in Fig. 2. Given the relatively wide spread of activity in the tilted dorsal-ventral dimension (∼1.5 mm) and the reported size of L2a in the dorsal-ventral dimension in coronal slices (Fortune & Margoliash, 1992; Vates *et al.*, 1996), we conclude that the BOLD activation that we measured also extends to the neighbouring subareas L1 and L3.

As illustrated in Fig. 3, the images obtained under urethane appear to have a second pole of significant BOLD signal found in the medial-ventral region. Based on its anatomical location, this second pole could be the medial-ventral subarea L within field L or the secondary auditory region NCM (Fortune & Margoliash, 1992; Vates *et al.*, 1996). The anatomical location of this second pole could also correspond to the location of the functional regions NA2a or the medial-ventral region of NA-L defined by Gehr and colleagues (Gehr *et al.*, 1999). A clear second pole (i.e. a separate area of significant activation) was found in only two of the four experiments performed under urethane anaesthesia. However, under both urethane and isoflurane, we also observed activation in this same region, not as a secondary pole but as a continuous extension of the activation observed in the primary auditory area. In summary, under all anaesthetics, we found activation centred around L2a and extending to L1, L3 and the dorsal region of L. Under urethane and isoflurane anaesthetic we found a larger area of activation that under medetomidine centred in the same region but that also extended into the medial-ventral region of L and potentially NCM. The secondary auditory regions CMM and CLM were not activated in the large majority of conditions, although significant pixels (but with relatively weak signal strength) were found in those regions in one experiment under isoflurane. BOLD responses were never found in song nucleus RA for any stimulus condition (including the BOS) or anaesthetic conditions (including urethane).

### Effect of anaesthesia

**Fig. 4:**
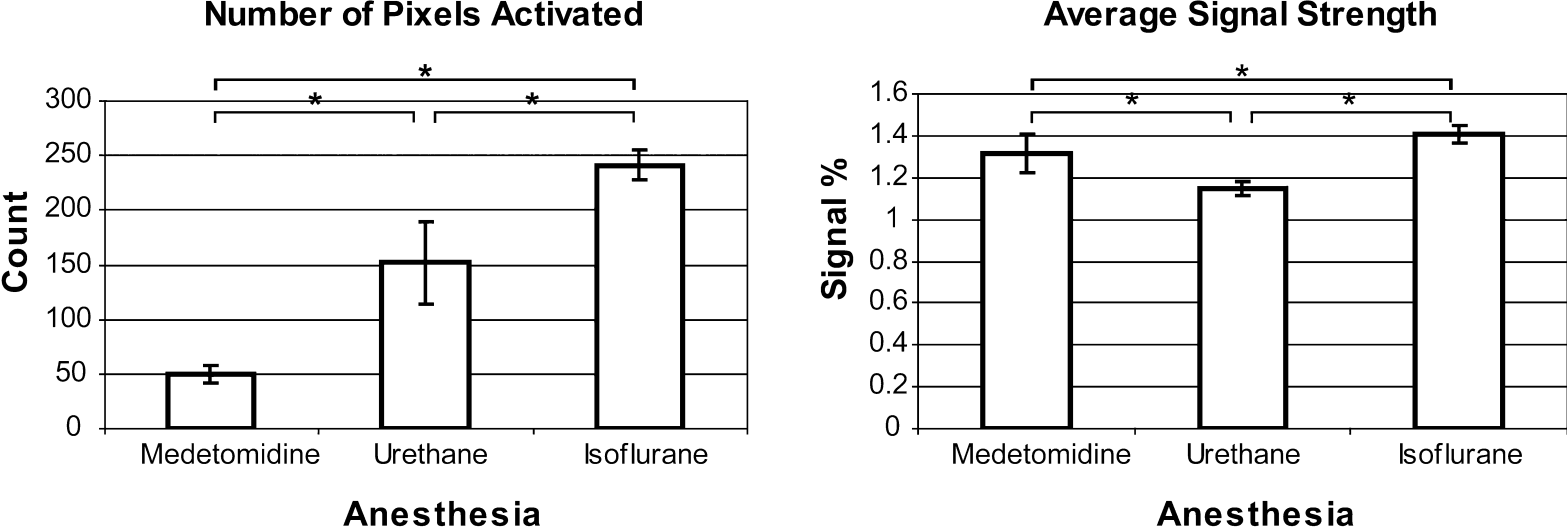
Anaesthesia and BOLD signal spread and strength. Left panel: the average total number of activated pixels under each anaesthetic condition. Each pixel is 97 × 97 µm. Right panel: the average strength of the signal (averaged across birds, pixels and all stimulus types) under each anaesthetic condition. The strength is measured as a percentage relative to background. The number of pixels and average strengths are calculated from all pixels showing significant BOLD activation. Significant differences are indicated by asterisks.

As illustrated in Fig. 3, the activated region was larger under urethane and isoflurane than under medetomidine. This observation was quantified in two ways. First, the area of activity in the primary auditory region was approximated by multiplying the medial-lateral extent with the dorsal-ventral extent. This area was greater for urethane (8.2 mm^2^) and isoflurane (6.8 mm^2^) than for medetomidine (2.6 mm^2^). The differences are statistically significant (one-way anova, *F*_2,13_ = 14.5; *P* = 0.001). Second, we more precisely measured the activated area by counting the number of significant pixels. The significant pixels were mostly found in the primary auditory regions, but the count included all pixels including those found in other regions. As shown in Fig. 4, the number of significantly activated pixels was the greatest for isoflurane, followed by urethane, with the least activated pixels for medetomidine. These differences were statistically significant (one-way anova, *F*_2,13_ = 17.1; *P* < 0.001).

The average signal strength (averaged across pixels, animals and stimulus types) appeared similar for the three anaesthetics as shown in Fig. 4, but the differences were significant (one-way anova, *F*_2,1874_ = 10.2; *P* < 0.001). The average signal strength in all significantly activated pixels was 1.15% for urethane, 1.3% for medetomidine and 1.4% for isoflurane. Peak signal strengths of up to 5%, such as those shown in Fig. 3, were commonly observed.

In order to compare responses under different anaesthetics without the potential confound of activity measured outside of the primary activation region, we defined a rectangular (ROI) of about 11 mm^2^ that was restricted to the large bilateral contiguous activated area centred around the primary auditory region L2a. All subsequent analyses reported below were done for this area only.

### Stimulus selectivity

We hypothesized that the auditory system of songbirds would be tuned to the characteristic spectral and temporal features of CON. Moreover, we hypothesized that distinct auditory regions would be more sensitive to spectral features, while other auditory regions would be more sensitive to temporal features. To test these hypotheses, we designed filtered song stimuli that preserved either the temporal (spectrally filtered) or spectral (temporally filtered) structure in the original song (see Materials and methods).

**Fig. 5:**
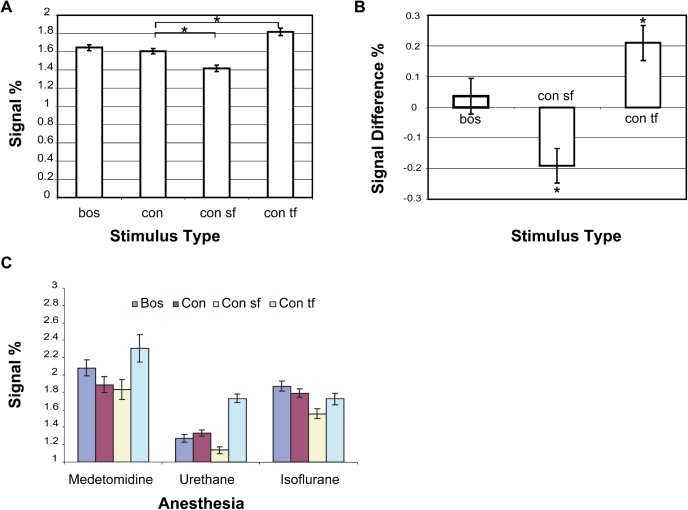
Stimulus selectivity.(A) The average responses (over all pixels) to the four different stimuli: bird's own song (BOS), conspecific song (CON), spectrally filtered CON (CON-sf) and temporally filtered CON (CON-tf), for the three different anaesthetic conditions (medetomidine, urethane and isoflurane).(B) Pair-wise differences in response between CON and the three other stimuli BOS, CON-sf and CON-tf.(C) The average responses (over all pixels) to the four different stimuli used in the experiment for medetomidine, urethane and isoflurane. Error bars show the 95% confidence interval. The average strengths are calculated only for the significantly activated pixels within the ROI. Significant differences are indicated by asterisks.

If the auditory system is tuned to the spectral and temporal structure found in natural CON, we would expect to find a decrease in activity to the synthetic song stimuli, which can be thought of as deteriorated song. Figure 5 shows the average responses (over all pixels in the ROI) to the four different stimuli used in the experiment, for all three different anaesthetic conditions separately (Fig. 5C) as well as averaged (Fig. 5A and B). A factorial anova reports a main effect of stimulus type (*F*_3,4228_ = 19.9; *P* < 0.001), a main effect of anaesthesia (*F*_2,4220_ = 42.71; *P* < 0.000001), and an interaction between anaesthetic and the selectivity for stimulus type (*F*_6,4220_ = 9.63; *P* < 0.000001). The main effect for stimulus showed one expected result and one, *a priori*, surprising result. As predicted from our hypothesis, the spectrally filtered synthetic songs elicited slightly smaller overall responses than the unfiltered song. As illustrated in Fig. 5B, the pair-wise difference in response between CON-sf and CON is negative and significant at the 0.1% level, after accounting for multiple comparisons (*t*_1057_ = −6.646851; *P* < 0.001/3). In other words, removing the spectral information from song, while preserving the temporal profile, led to smaller overall BOLD responses. This would be expected if neurons are not only tuned to temporal modulations in song but also to spectral structure. Removing the spectral structure by replacing it with broadband coloured noise should lead to decreased firing rates and reduce BOLD responses. By contrast, we also found an *a priori* unexpected effect. The response to the temporally filtered song was on average greater than to the unfiltered song. The pair-wise difference in response between CON-tf and CON is positive and highly significant (*t*_1057_ = 6.788531; *P* < 0.001/3). In this case, it appears that removing the temporal structure in song led to an increase in response. This unexpected result will be discussed later. Finally, in terms of main effect for stimulus type, we did not observe any overall differences between the responses to BOS and CON (*t*_1057_ = 1.111753; *P* = 0.266497).

As illustrated in Fig. 5C, the main effect of anaesthesia in the ROI is due to a BOLD signal that was largest under medetomidine, intermediate under isoflurane and smallest under urethane. Note that the average signal strengths and their order within the ROI for the different anaesthetics are somewhat different than those reported for all the significantly activated pixels (see Fig. 4 and above). An interaction was found between anaesthetic and stimulus. This interaction effect was principally caused by the response to the temporally filtered song. This response was larger under urethane and medetomidine (as described above), while it was slightly depressed under isoflurane. The pair-wise difference in response between CON-tf and CON is highly significant under urethane (*t*_351_ = 15.867302; *P* = 0.000000), but showed no significance under medetomidine (*t*_271_ = 1.472577; *P* = 0.142026) or isoflurane (*t*_433_ = −1.882524; *P* = 0.060435) after accounting for multiple comparisons (*P* < 0.05/3). On the other hand, the response to the spectrally filtered song was depressed under all conditions. The pair-wise difference in response between CON-sf and CON is significant under all three anaesthetic conditions (medetomidine: *t*_271_ = −3.204164; *P* = 0.001517; urethane: *t*_351_ = −2.478455; *P* = 0.013665; isoflurane: *t*_433_ = −5.754848; *P* = 0.0000000). This effect will be discussed later, but the immediate conclusion from these results is that the type of anaesthesia affects the temporal tuning of auditory responses more than their specificity to acoustic spectral structure.

### Functional auditory regions

The second part of our hypothesis was that we would find specialized auditory brain regions for processing spectral structure vs temporal structure. The spatial resolution of the structural MR images was sufficient to coarsely delineate the primary auditory region L2a as well as to find the boundaries between the nidopallium and mesopallium, and thus to delineate CLM and CMM. However, because the principal auditory evoked BOLD response was in field L and centred around L2a, we chose to perform a regional analysis by dividing the auditory regions into four quadrants: the right and left hemisphere, and the ventral and dorsal regions of field L. Based on our measurements and the comparison to previously published anatomy (see above), the dorsal quadrants include the dorsal part of L2a and L1 and potentially L2b, while the ventral quadrants include the ventral part of L2a, L3 and, in some experiments, the medial-ventral part of subregion L as well as potentially NCM.

**Fig. 6:**
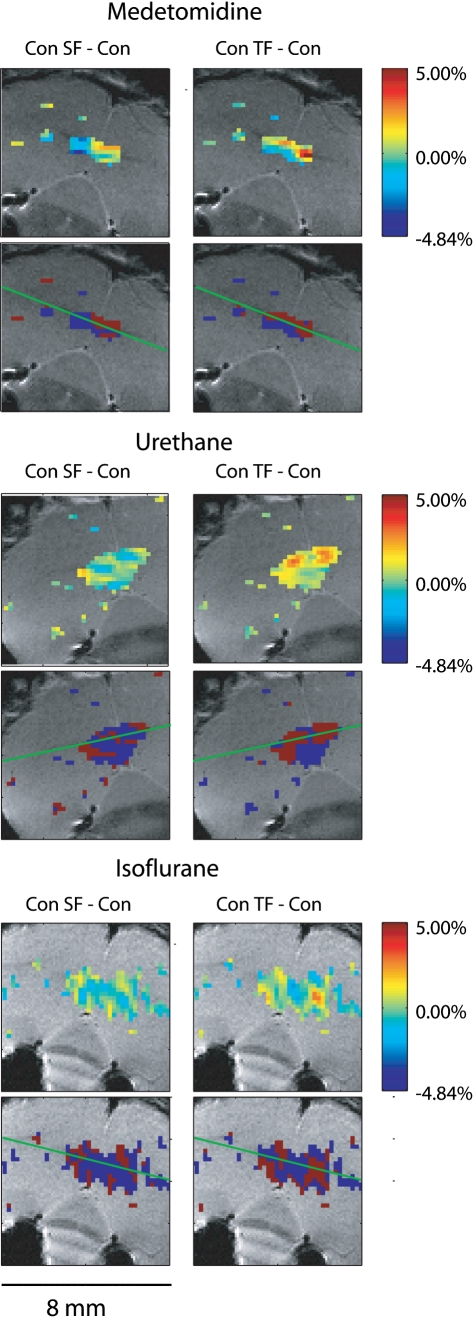
Example of regional selectivity. The BOLD difference map between spectrally filtered song and normal song (left column) and between temporally filtered song and normal song (right column) are shown for all three anaesthetic conditions for one example bird. The overall BOLD signal and significance maps for this bird were shown in Fig. 3. The colour maps on the first rows show the difference in responses in terms of percentage of the average signal and not in terms of percentage of maximum possible difference. In other words, because the signals are all below 5% (see Fig. 3), a difference of 5% would correspond to 100% modulation. The red/blue maps in the second row show the regions where the difference is above (red) and below (blue) the mean difference, which in most cases is very close to zero. The green line shows the dorso-ventral axis. These examples are typical of the data. In the medetomidine and urethane plots, one can observe the depressed response for both the temporally filtered and spectrally filtered songs in the ventral areas relative to the dorsal areas. One can also observe the overall higher responses to the temporally filtered song relative to conspecific song. The difference maps obtained under isoflurane show less selectivity but are not inconsistent with the results obtained on average. CON, conspecific song; CON-sf, spectrally filtered CON; CON-tf, temporally filtered CON.

**Fig. 7:**
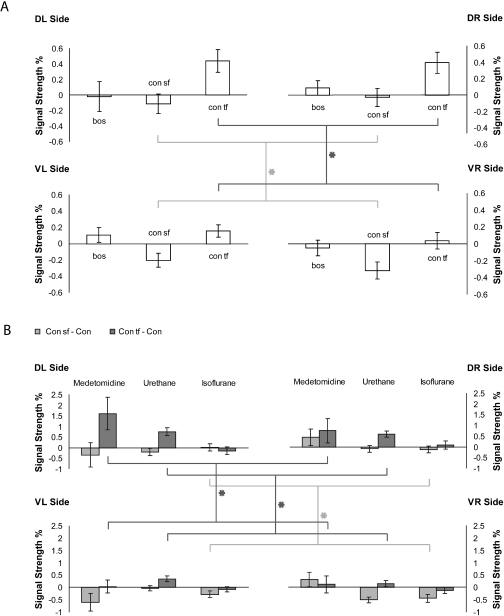
Stimulus and regional selectivity.(A) The average BOLD signal for the synthetic songs and for the bird's own song (BOS) relative to the BOLD signal found for conspecific song (CON) is shown for the four quadrants: left vs right hemisphere, and ventral vs dorsal region.(B) The BOLD responses to the spectrally (CON-sf) and temporally (CON-tf) filtered songs relative to the BOLD signal for CON found in the dorsal and ventral regions is shown for the three anaesthetic conditions. Error bars show the 95% confidence interval. The average strengths are calculated only for the significantly activated pixels within the ROI. Significant differences are indicated by asterisks.

Figure 6 illustrates the typical difference maps that we obtained when we compared the responses to the filtered song from those to the normal song: a depressed response for both the spectrally and temporally filtered version of the song is found in the ventral region relative to the dorsal region and the temporally filtered song elicits stronger overall responses. Figure 7 quantifies these results for all three anaesthetic conditions averaged together (Fig. 7A) and for each anaesthetic condition analysed separately (Fig. 7B). Figure 7A shows the average BOLD signal in the four quadrants relative to the BOLD signal found for CON. A two-way anova (dorsoventral and right–left) for each pair-wise comparison was performed and the *P*-values were interpreted after a Bonferroni correction for multiple comparisons. There was no main effect of laterality (right–left) for any of the three stimuli (BOS: *F*_1,1054_ = 0.13; *P* = 0.72; CON-sf: *F*_1,1054_ = 0.06; *P* = 0.8; CON-tf: *F*_1,1054_ = 1.62; *P* = 0.2). There was also no main effect in the dorsoventral dimension for BOS (BOS: *F*_1,1054_ = 0; *P* = 0.95). However, there was a strong effect along the dorsoventral dimension for the CON-sf and CON-tf stimuli (CON-sf: *F*_1,1054_ = 9.98; *P* = 0.0016; CON-tf: *F*_1,1054_ = 24.62; *P* < 0.000001): the depressed responses to the CON-sfs were found solely in the ventral regions, and the responses to the CON-tfs were significantly increased in the dorsal regions. The same statistical analysis was performed for each anaesthetic condition. Figure 7B shows BOLD responses to the spectrally and temporally filtered songs relative to the BOLD signal found for CON found in the dorsal and ventral regions for the three anaesthetic conditions. As in the combined data, a depressed response for the spectrally filtered song in the ventral regions relative to dorsal regions was observed under all anaesthetic conditions. This effect was significant under isoflurane (*F*_1,430_ = 13.52; *P* = 0.0003) and showed a similar trend under medetomidine and urethane, although the results were not significant (urethane: *F*_1,348_ = 4.41; *P* = 0.037; medetomidine: *F*_1,268_ = 0.49; *P* = 0.48). In addition, we found that the temporally filtered song also had weaker responses in the ventral areas relative to the dorsal areas under urethane (*F*_1,348_ = 25.61; *P* < 0.000001) and medetomidine (*F*_1,268_ = 23.59; *P* < 0.000001), but showed no dorsoventral difference under isoflurane (*F*_1,430_ = 0.92; *P* = 0.338). Thus, the difference in responses to the temporally filtered song under isoflurane relative to urethane and medetomidine affected not only the overall responses as illustrated in Fig. 5, but also the relative strength of the responses in the ventral vs dorsal regions. Finally, we did not find reliable differences in responses between BOS and CON across these four different regions for any anaesthetic.

## Discussion

### BOLD responses in avian auditory forebrain

Our study showed robust BOLD activity in the avian primary auditory forebrain in response to CON as well as to spectrally and temporally filtered versions of the song. Based on specific landmarks that are clearly seen in the structural MR image and on our measurements of the spread of the activity, we deduced that BOLD activity was present in most of field L: the peak of BOLD activity was clearly in L2a, the primary thalamo-recipient region, but the activity spread to neighbouring subareas L2b and L1 on the dorsal side, L3 on the ventral side and L on the medial-ventral side. It is possible that the BOLD signal in the ventral-medial regions also reflected neural activity in the secondary auditory area NCM. However, as the actual border between L3 or L2b/L and NCM cannot be seen in the structural MR image and is not precisely reported in more detailed histological studies (Fortune & Margoliash, 1992), we are not able to make such a claim conclusively.

The lateral extent of activity within field L (and in particular L2a) corresponds well to the robust auditory responses measured in neurophysiological studies (Grace *et al.*, 2003). On the other hand, we noted that there was an absence of BOLD signal in secondary auditory regions CLM and CMM (and potentially NCM), while the presence of auditory neurons in those areas is well established (Sen *et al.*, 2001; Gentner & Margoliash, 2003; Grace *et al.*, 2003). A possible explanation for this discrepancy might be differences in the strength of the neural response, in the density of auditory neurons and/or in the difference between afferent vs efferent response profiles in the two areas. The strength of the neural responses in field L for single units (measured with a *z*-score) is approximately twice as large as that measured for CLM neurons (Grace *et al.*, 2003). There is also a higher density of neurons in L2a than in the neighbouring nidopallium or mesopallium (Fortune & Margoliash, 1992), and a higher proportion of those neurons show auditory activity (Grace *et al.*, 2003). The evoked auditory activity in CLM and CMM might therefore not be sufficiently different from background to elicit a BOLD signal (Norup Nielsen & Lauritzen, 2001; Sheth *et al.*, 2004). Another possible confound is that the neurophysiological studies in field L and CM are measuring the spiking activity of either projection neurons or interneurons, while the BOLD signal could mostly reflect synaptic activity and therefore only input spikes from afferent neurons or interneurons (Logothetis *et al.*, 2001; Lauritzen & Gold, 2003; Kayser *et al.*, 2004). The observed BOLD signal might therefore be a reflection of the strong and focused thalamic input into L2a, and from L2a to L1 and L3. The more diffuse input to CMM and CLM could be undetectable (Vates *et al.*, 1996).

Along similar lines, we did not find any BOLD activity in RA, although robust auditory responses to the BOS under urethane anaesthetic have been reported in neurophysiological experiments (Doupe & Konishi, 1991; Vicario & Yohay, 1993; Kimpo *et al.*, 2003). Despite the robustness of these auditory responses, they are found on top of a relatively high background activity that presumably requires a constantly high metabolic rate. A saturation (or ceiling) effect in the BOLD transfer function could then dampen the stimulus-driven response modulations. The transfer functions between summed neural response and BOLD have been reported to be either expansive, linear (Hewson-Stoate *et al.*, 2005) or compressive (Lauritzen & Gold, 2003), depending on stimulus conditions such as baseline stimulus intensity and stimulus dynamics. A compressive function would explain the lack of significant BOLD signal differences observed here. We are therefore reporting the presence of two brain regions where there is a discrepancy between reported neural activity and the BOLD signal that we recorded, potentially because the neural activity was too much below threshold in one case, and above saturation in the other case, to obtain robust changes in cerebral blood flow. Finally, it should be noted that in fMRI studies the sound is presented over background noise due to the on/off switching of magnetic field gradients, whereas in neurophysiological studies the sounds are presented in a quiet environment.

### Effect of anaesthetic

Our study also found different responses under different anaesthetic conditions. One major difference was a larger area of activation measured under isoflurane and urethane relative to medetomidine. These three anaesthetics work on different neurotransmitter systems. Medetomidine, a non-narcotic sedative and analgesic, is a potent α2-adrenoreceptor agonist that produces sedation and analgesia. Urethane appears to modulate all neurotransmitter-gated ion channels, including the potentiation of inhibitory γ-aminobutyric acid (GABA) and glycinergic synapses and the depression of excitatory glutamatergic synapses (Hara & Harris, 2002). Isoflurane exerts its greatest effects on GABAergic synapses, causing a marked increase in total charge transfer through the inhibitory postsynaptic current. At glutamatergic synapses, isoflurane has smaller effects, but it nonetheless significantly reduces the total charge transfer through the excitatory postsynaptic current (reviewed in Richards, 2002). Based on these differences in mechanisms of action, one might have predicted that medetomidine would have the smallest effect on neural activity as it is acting on a neuromodulator system, whereas urethane and isoflurane act on the major excitatory and inhibitory ionotropic channels.

Our results, however, are not consistent with this simple prediction as we found the smallest area of BOLD activity under medetomidine anaesthesia. On the other hand, the peak signal strength was centred around L2a under all three anaesthetic conditions, and the major difference was in the spread of activity to auditory subregions within field L. It is therefore possible that adrenergic modulation selectively affected these secondary auditory areas. Consistent with that explanation, it has been shown that noradrenergic terminals are found throughout the avian auditory and vocal systems (Mello *et al.*, 1998), and that α-adrenergic receptor blockade abolishes song-induced ZENK induction in the secondary auditory area NCM of zebra finches (Ribeiro & Mello, 2000). In medetomidine-anaesthetized starlings, stimulus-specific fMRI responses were previously revealed in the NCM, but this drug was given together with a much larger dose of ketalar (Van Meir *et al.*, 2005).

We also found an interaction between anaesthesia and stimulus. Relative to the responses to the unfiltered song, the responses to the temporally filtered song were depressed under isoflurane, while they were enhanced under medetomidine and urethane. The enhancement of baseline inhibitory activity under isoflurane might explain this result. The neural response in higher auditory areas to sustained sounds such as the temporally filtered version of song are characterized by a strong onset response followed by a much smaller sustained response (Grace *et al.*, 2003). The phasic-sustained shape of such auditory responses in the midbrain has been shown to be shaped in great part by the inhibitory circuitry (e.g. Yang & Pollak, 1994; Le Beau *et al.*, 1996). Therefore, it is highly probable that the disruption of the inhibitory circuitry would affect these responses. On the other hand, whether enhancing baseline inhibition would decrease or increase the overall response to sustained sounds (temporally filtered song) relative to more phasic sounds (normal song) remains to be tested. Moreover, the enhancement of the BOLD response to the temporally filtered version of song relative to normal song under urethane and medetomidine also requires an explanation as this result was not expected based on previous neural recordings. Indeed, although the neural spiking responses to the temporally filtered song are expected to be more sustained, the sum of the phasic spiking responses to each syllable obtained for the normal song is expected to yield equal or higher firing rates. This result was clearly observed in neural recordings under urethane anaesthesia in the song nucleus HVC (Theunissen & Doupe, 1998), and a similar effect was found when responses to song were compared with responses to other acoustically matched sustained sounds (Grace *et al.*, 2003; Hsu *et al.*, 2004). The enhancement in BOLD response to the temporally filtered version of the song could thus be due to greater subthreshold activity during the temporally filtered song (reflecting potentially higher inhibition) or to the non-linear dynamics of the BOLD transfer response. Similar findings have been observed in the cat visual cortex where the spiking response to natural images is much greater than the spiking response to visual gratings, but where the opposite relationship is found in the BOLD signal (Kayser *et al.*, 2004).

In summary, we have so far discussed differences between neural activity and BOLD response as well as the complicated effects of anaesthesia. Although this analysis illustrates some of the limitations of the fMRI method, it also shows that the avian auditory and vocal system could also serve as a powerful model system to better understand the nature of the transfer function between neural activity and BOLD responses. Moreover, these results show that fMRI in combination with electrophysiology could be used as a tool to study the role of inhibitory circuitry and neuromodulator systems in high-level sensory processing. For this purpose the use of the songbird system where substantial neurophysiological, anatomical, genetic and behavioural studies are carried out (Zeigler & Marler, 2004) could play an important role.

### Specialized auditory regions for spectral and temporal processing

The major goal of our study was to determine whether differential regions of the auditory system would be specialized for the spectral vs temporal structure found in behaviourally relevant complex natural sounds. Significant effects along the dorso-ventral axis were found for either the spectrally filtered or temporally filtered (but not both) within different anaesthetics. In this analysis, the most consistent result is that the ventral regions of the zebra finch auditory forebrain are more affected by spectral filtering than the dorsal regions. Responses to temporally filtered song are more difficult to interpret. The responses were, on average, greater than those for normal song, potentially because of the nature of the BOLD transfer function and the effect of anaesthesia as described above. But, similarly as for the spectrally filtered song (but significant only under a different anaesthetic condition), the responses to temporally filtered song in the ventral areas were smaller than those found in the dorsal areas. Our results therefore show that the dorsal regions of field L are in fact responsive to spectral structure as this is the structure that remains in the temporally filtered song. However, for sounds that have a natural temporal pattern, only the ventral regions showed a depressed response to sounds lacking the natural spectral patterns. Thus, the avian ventral auditory regions might be specialized to process the spectral information found in complex natural sounds or, more specifically, might be more specialized for CON processing, as this spectral sensitivity is correlated with a temporal sensitivity. We deduced that this ventral auditory region corresponds to the ventral part of L2a as well as L3 and the medial-ventral part of subarea L, whereas the dorsal region corresponds to the dorsal part of L2a as well as L1 and L2b and the dorsal part of subarea L. Our study would therefore show the first functional difference between L1 and L3, which have otherwise been described as mostly symmetric in terms of their afferent connectivity (Vates *et al.*, 1996) as well as in terms of the neurophysiological properties of their neurons (Lim & Kim, 1997; Sen *et al.*, 2001). The idea that L3 could be specialized for spectral processing or song processing is congruent with its place in the auditory forebrain pathways: L3 is found between the primary thalamic-recipient L2a and the secondary auditory area NCM (Vates *et al.*, 1996). NCM, in turn, has been shown in a series of experiments to be key for the representation of familiar CONs (reviewed in Bolhuis & Gahr, 2006).

Our result also supports the idea that the primary auditory forebrain region field L could be divided not only into anatomical areas but also in terms of functional areas (Gehr *et al.*, 1999; Cousillas *et al.*, 2005). Our ventral subdivision overlaps with the functional auditory areas NA2a and NA3 defined by Gehr and colleagues based primarily on frequency mapping (Gehr *et al.*, 1999). In that report, it was observed that responses to pure tones in the dorsal areas of the auditory neostriatum (NA2b and NAL) were more phasic than those in the ventral areas (NA2a and NA3). That observation is consistent with a ventral area that would be more specialized for spectral processing relative to the dorsal area. It should also be noted that our study did not systematically investigate other potential functional partitions, such as divisions along the medial/lateral axis or the rostral/caudal axis. Further neurophysiological and brain mapping studies will be required to determine the degree of specialization and the precise anatomical location of these still putative functional regions. Our study predicts that in such studies, a higher number of auditory neurons specialized for spectral processing will be found in the ventral region.

The comparison with human and other primate findings is still tenuous at this point. Most human studies have implicated higher auditory or association cortical regions as critical for music perception. For example, the lateral Heschl's gyrus, a secondary region that is anterolateral to the primary auditory cortex, appears to be involved in the perception of pitch (Griffiths, 2003). Similarly, single neurons in a homologous region in the marmoset have complex response properties that can be explained as a representation of the pitch percept (Bendor & Wang, 2005). While our study also points to an auditory brain region that could be specialized for spectral information, it was found within the primary auditory forebrain area and not in a secondary auditory region. It is possible that secondary regions in the birds show further specialization but a BOLD signal was not robustly observed in these areas under anaesthesia. Finally, we also failed to find any lateralization effects. One robust finding in both lesion and brain imaging studies in humans is that cortical function is lateralized. In terms of music perception, rhythm appears to be more localized to the left hemisphere, while timbre and melody are more localized to the right hemisphere (Zatorre, 2001; Kohlmetz *et al.*, 2003; Di Pietro *et al.*, 2004; Murayama *et al.*, 2004). Human speech as well as monkey vocalizations appear to be preferentially processed on the left cortical hemisphere (Poremba *et al.*, 2004). Similarly, lesion experiments suggest that songbirds, including zebra finches, must have neural systems lateralized for both vocal production and perception (Cynx *et al.*, 1992; Williams *et al.*, 1992; Floody & Arnold, 1997; George *et al.*, 2006). The discrepancy between those findings and our study might also be due to our failure to measure BOLD responses in higher auditory areas that might be more lateralized in their function than the primary auditory region. The lateralization effect might also come into play only for higher order perceptual attributes. For example, melody perception might be lateralized, whereas pitch perception might not be (Bendor & Wang, 2006). Similarly, in songbirds one can postulate that spectral and temporal feature extraction is not lateralized while song recognition is. Further imaging and neurophysiological experiments in songbirds can elucidate the nature of the auditory network that underlies such complex auditory tasks as recognizing particular songs and, by comparison and analogy, recognizing musical melodies or spoken sentences in humans.

## Abbreviations

BOLD: blood oxygen level-dependent
BOS: bird's own song
CLM: caudal lateral mesopallium
CM: caudal mesopallium
CMM: caudal medial mesopallium
CON: conspecific song
CON-sf: spectrally filtered CON
CON-tf: temporally filtered CON
fMRI: functional magnetic resonance imaging
FOV: field of view
GABA: γ-aminobutyric acid
GE: gradient-echo
MR: magnetic resonance
NCM: caudal medial nidopallium
RA: robust nucleus of the arcopallium
RF: radio-frequency
ROI: region of interest
SPL: sound pressure level
TE: echo time
TR: repetition time.
